# The potential value of Notch1 and DLL1 in the diagnosis and prognosis of patients with active TB

**DOI:** 10.3389/fimmu.2023.1134123

**Published:** 2023-03-28

**Authors:** Jinling Xie, Yinzhong Chen, Shihao Chen, Huaquan Long, Weijian Zhang, Guoan Liu

**Affiliations:** Xinhui District People’s Hospital, Affiliated with the Southern Medical University, Jiangmen, China

**Keywords:** Notch1 receptor, DLL1 ligand, ROC curve, active tuberculosis (TB), biomarkers

## Abstract

**Objectives:**

The Notch signaling pathway has been implicated in the pathogenesis of active tuberculosis (TB), and Th1-type cell-mediated immunity is essential for effective control of mycobacterial infection. However, it remains unclear whether Notch signaling molecules (Notch1, DLL1, and Hes1) and Th1-type factors (T-bet and IFN-γ) can serve as biomarkers for tracking the progression of active TB at different stages along with peripheral blood white blood cell (WBC) parameters.

**Methods:**

A total of 60 participants were enrolled in the study, including 37 confirmed TB patients (mild (n=17), moderate/severe (n=20)) and 23 healthy controls. The mRNA expression of Notch1, DLL1, Hes1, T-bet and IFN-γ in the peripheral blood mononuclear cells (PBMCs) of the subjects was measured by RT-qPCR, then analyzed for differences. Receiver Operating Characteristic curve (ROC) was used to assess the effectiveness of each factor as a biomarker in identifying lung injury.

**Results:**

We found that mRNA expression levels of Notch1, DLL1, and Hes1 were upregulated in active TB patients, with higher levels observed in those with moderate/severe TB than those with mild TB or without TB. In contrast, mRNA levels of T-bet and IFN-γ were downregulated and significantly lower in mild and moderate/severe cases. Furthermore, the combiROC analysis of IFN-γ and the percentage of lymphocytes (L%) among WBC parameters showed superior discriminatory ability compared to other factors for identifying individuals with active TB versus healthy individuals. Notably, Notch pathway molecules were more effective than Th1-type factors and WBC parameters in differentiating mild and moderate/severe cases of active TB, particularly in the combiROC model that included Notch1 and Hes1.

**Conclusions:**

Our study demonstrated that Notch1, Hes1, IFN-γ, and L% can be used as biomarkers to identify different stages of active TB patients and to monitor the effectiveness of treatment.

## Introduction

Tuberculosis (TB) is a widespread infectious disease caused by Mycobacterium tuberculosis, with the lungs being the most commonly affected organ. Despite numerous efforts to combat the disease, it persists as a serious global public health challenge. The World Health Organization’s Global TB Report 2021 (1) showed that prior to the coronavirus (COVID-19) pandemic, TB was the leading cause of death from a single infectious agent, surpassing even HIV/AIDS. Although extensive research has been conducted on the pathogenesis of TB, there is currently no cost-effective and reliable hematological method available for monitoring the progression of active TB and the effectiveness of treatment.

Upon infection with TB, the host mounts both adaptive and innate immune responses aimed at eliminating the bacteria. Lymphocytes are key immune cell types involved in this process. Previous studies have shown that IFN-γ secretion by Th1 cells (a type of T-cell subset) is beneficial for activating macrophages and mononuclear cells to control TB infection in the early stages, enhancing host autophagy and bacterial clearance (2–4). However, the persistent secretion of IFN-γ by T cells in patients with latent TB can lead to the development of active TB within four years (5). This suggests that a strong association between IFN-γ secretion by Th1 cells and disease progression of TB.

Of note, the role of Notch signaling in disease has received considerable attention in recent years. Studies have reported that this pathway can protect colonic epithelial cells from proinflammatory responses (6), promote immune molecules expression and exacerbate cytokine disorders (7), and also involves in Th1 differentiation and IFN-γ secretion (8, 9). The Notch signaling pathway is a highly conserved pathway that mediates cell-cell communication through various Notch signaling molecules. These molecules are present ubiquitously on the cell surface and play a crucial role in determining the fate of the cell. In mammals, the binding of the Notch receptor (Notch1-4) to Notch ligand proteins (Delta-like 1, 3, 4, Jagged1, and Jagged2) leads to the activation of CSL transcription factors (CBF-1, Suppressor of Hairless, Lag1) and downstream target genes (such as Hes1), forming signal transduction pathways. For example, Notch signaling was activated after binding of Notch receptor with ligand DLL1, which influences the direction of cell differentiation and regulates cell fate (10, 11).

In addition, recent studies have confirmed that Notch signaling is involved in the progression of active TB (12–14). Blockade of Notch signaling by DAPT restores theTh1/Th2 imbalance in TB patients (13). DLL1 levels in cerebrospinal fluid (CSF) and serum of patients with tuberculous meningitis (TBM) were higher than those in the bacterial meningitis, viral meningitis/encephalitis and nondiagnosed groups, indicating that DLL1 may be a new biomarker for TBM diagnosis (12). Based on these findings, we hypothesized that Notch signaling molecules (Notch1, DLL1 and Hes1) and Th1-type response factors (T-bet and IFN-γ), along with differences in white blood cell (WBC) parameters may serve as potential biomarkers for predicting disease progression and monitoring therapeutic effect in patients with active TB.

In the current study, the mRNA levels of Notch1, Hes1 and DLL1 were upregulated in PBMCs from active TB patients, while the expression of T-bet, and IFN-γ were downregulated. IFN-γ was superior to Notch pathway molecules in identifying active TB patients from healthy controls, particularly the model combined with L% had the best discriminatory power. Interestingly, Notch pathway molecules could better differentiate mild and moderate/severe cases of active TB than Th1-type factors and WBC parameters. Especially, the combiROC model with Notch1 and Hes1 was more effective. These may provide novel insights into the use of Notch signaling molecules as biomarkers of progression and efficacy in active TB.

## Materials and methods

### Study subjects

Study participants were obtained at Xinhui District People’s Hospital affiliated with Southern Medical University from September 2021 to July 2022, and were aged 18 years or older. The patients with confirmed active TB who had not yet started anti-TB treatment and a control group of apparently healthy individuals from the same hospital’s medical examination center underwent physical examinations. This study had been approved by the Ethics Committee of Xinhui District People’s Hospital affiliated with Southern Medical University, Jiangmen (Medical Research Ethics Audit 2019 No. 027), and written informed consent was obtained from the participants.

Individuals enrolled in this study were described in detail as follows. Negative chest x-ray and IFN-γ release assay results further confirmed apparently healthy control subjects. Patients with active TB were diagnosed on the basis of clinical manifestations, positive results of at least one laboratory test (TB DNA test, acid-fast bacilli smear test, IFN-γ release test), and characteristics of TB on chest radiograph, meeting the diagnosis criteria of Tuberculosis WS288-2017 (15). Then, they were classified as mild, moderate, and severe groups based on the lung injury and symptoms. In mild cases, symptoms are mild and imaging shows mainly plaques, nodules, and striae or tuberculomas or isolated cavities. In moderate cases, symptoms may include cough, sputum production, shortness of breath, or low-grade fever. Imaging may show scattered lesions of low to moderate density in one or both lungs, or even fusion, but the area of high-density lesions does not exceed one-third of the volume of a lung. In severe cases, symptoms include dyspnea, hemoptysis, or hyperthermia. Imaging may show lobar infiltrates, caseous pneumonia, multiple cavitations, and bronchial dissemination, even extrapulmonary dissemination occurs. The study excluded individuals who were either taking antibiotics or had medical conditions that could potentially affect the study outcomes. These medical conditions included severe heart disease, significant liver disease, active infectious diseases, hematologic disorders, autoimmune diseases, and receiving immunotherapy.

### Blood sample preparation

​Approximately 7 mL of venous blood was collected from TB patients and healthy individuals *via* venous puncture. Blood samples were drawn into tubes containing EDTA-K2 (BD Vacutainer, New Jersey, USA). Of the 7 ml, 5 ml was used for the peripheral blood mononuclear cells (PBMCs) isolation, and the remaining 2 ml was used to register the complete blood count. PBMCs were separated by Ficoll-Hypaque density gradient centrifugation (TBD, Tianjin, China), which was centrifuged at 400 x g for 30 min at 20^∘^C.

### Complete blood count

Blood samples were analyzed for peripheral blood cell count using the Sysmex XN2000 automated hematocytometer (Sysmex, Tokyo, Japan). It provides analysis of peripheral blood cell components, cell volume, cell percentage and other classification parameters. Among these parameters, the number of total white blood cells, the percentage and the number of granulocytes, lymphocytes and monocytes are widely used in the monitoring of infectious diseases. Complete blood count is performed under strict quality control procedures. These include daily high and low internal quality control, bi-weekly quality control and annual quality assurance as the important parts of the Clinical Laboratory QC Program.

### RNA extraction and RT-PCR

According to the manufacturer’s protocol, the extraction of total RNA of PBMCs from individuals was used Trizol method (TRizol reagent, Takara Biotechnology, Dalian, China) and treated with RNase-free DNase (Solarbio, Beijing, China). And then reverse transcription was accomplished by a High-Capacity cDNA Reverse Transcription synthesis kit (Vazyme, Nanjing, China).

### Quantitative real-time PCR

Quantitative real-time PCR was performed using SYBR Green PCR mixture (Vazyme Nanjing, China) to validate the differential mRNA levels of Notch1, Hes1, DLL1, T-bet and IFN-γ under the following conditions: 45 cycles of 95°C for 30 seconds, 95°C for 10 seconds and 60°C for 40 seconds in an Applied Biosystems instrument. The human primer sequences were shown in **Table 1**. The relative fold change in mRNA expression of all genes was measured by the 2^−ΔΔCt^ method (16) using β-actin as the internal reference gene, according to the following calculation: ΔΔCt = experiment group (Ct target − Ct internal reference) − control group (Ct target − Ct internal reference). Means and SDs were computed from triplet datasets.

**Table 1:** Human primer sequences used in real- time PCR.

Gene	Human primer sequences 5’-3’
Notch1	F:5’-CACTGTGGGCGGGTCC-3’
	R:5’-GTTGTATTGGTTCGGCACCAT-3’
Hes1	F:5’-CGTGTCTCCTCCTCCCATT-3’
	R: 5’-GAGAGGTAGACGGGGGATTC-3’
DLL1	F:5’-GACGAACACTACTACGGAGAGG-3’ R:5’-AGCCAGGGTTGCACACTTT-3’
T-bet	F: 5’-GGATGCGCCAGGAAGTTTCA-3’ R: 5’-GACTGGAGCACAATCATCTGGG-3’
IFN-γ	F:5’-GTGTGGAGACCATCAAGGAAGACA-3’ R:5’-TTGGACATTCAAGTCAGTTACC-3’
β-actin	F:5’-CATGTACGTTGCTATCCAGGC-3’ R:5’-CTCCTTAATGTCACGCACGAT-3’

### Statistical analysis

Statistical analysis and graphs were performed by R language (mainly R package GGploT2 [version 3.3.3]) (used for visualization) and GraphPad Prism 6.0 (GraphPad Software, La Jolla, CA, USA; www.graphpad.com). Categorical variables were shown as number (%), and analyzed by χ^2^ test. Continuous variables were shown as mean ± SD and analyzed by Student’s t-test if normally distributed, or as median (interquartile range) and analyzed by Mann-Whitney U test otherwise. For non-normally distributed independent samples, Spearman method correlation analysis was used for assessing the relationship between two genes. One-way analysis of variance (ANOVA) and Kruskal-Wallis’s test were used for multivariate comparisons among groups. ROC curve analysis was used to evaluate the diagnostic efficiency of each factor. Differences were considered statistically significant if p-value was < 0.05. ∗*p* < 0.05; ∗∗*p* < 0.01; ∗∗∗*p* < 0.001; ∗∗∗∗*p* < 0.0001, ns, not significant.

## Results

### The percentage of lymphocytes in peripheral blood of active TB patients were lower than those of healthy controls

A total of 60 participants were enrolled in this study, of which 23 healthy individuals were negative controls and 37 TB patients were in the positive group. The main clinical information of all participants was shown in **Table 2**. The classification parameters of the WBC were obtained through the complete blood count conducting with the Automatic Hematology Analyzer Sysmex XN2000. No significant difference was observed in age, sex, total WBC count, neutrophil count, monocyte count and percentage (M%) between the positive and healthy groups. However, the percentage of lymphocytes (L%) was lower in the TB patients than that in healthy controls, while the percentage of neutrophils (N%), the ratio of neutrophils/lymphocytes (N%/L%), and the ratio of monocytes/lymphocytes (M%/L%) in TB individuals were higher than in the healthy controls. These results confirmed that the lymphocytes were more attrition in the progression of active TB compared to the healthy controls, which was similar with the conclusions of previous studies (17, 18). The increase in N% was likely due to a decrease in L%, as we term this phenomenon a “increased passivity”. This is because neutrophils, lymphocytes, and monocytes are the main components in peripheral blood leukocytes, with the normal reference range for N% being 50%-70%, and L% being 20%-40%. However, our study showed a true decrease in both percentage and absolute number of lymphocytes.

**Table 2:** Characteristics and white blood cell classification parameters of active TB patients and healthy individuals enrolled in this study.

Characteristic	Control group	Active TB	*P-value*
Numbers	23	37	
Age (years), mean ± SD	53.52 ± 15.94	62.3 ± 16.52	ns
Gender (*n* [%])			
female	9 (15%)	6 (10%)	ns
male	14 (23.3%)	31 (51.7%)	ns
White blood cells (E+09/L), median (IQR)	7.72 (7.15, 8.7)	7.97 (5.36, 11.59)	ns
Neutrophils (E+09/L), median (IQR)	4.34 (3.83, 5.56)	5.98 (3.52, 8.74)	ns
Percentage of Neutrophils (N%) mean ± SD	57.73 ± 6.76	72.25 ± 13.71	***
Lymphocytes (E+09/L), mean ± SD	2.71 ± 0.72	1.27 ± 0.60	****
Percentage of lymphocytes (L%) median (IQR)	35.15 (29.82, 37.65)	15.3 (7.95, 27.25)	***
Ratio of Neutrophils/lymphocytes (N%/L%), median (IQR)	1.65 (1.42, 2.02)	4.95 (2.29, 8.9)	***
Monocytes (E+09/L), mean ± SD	0.47 ± 0.1	0.55 ± 0.31	ns
Percentage of monocytes (M%) mean ± SD	5.96 ± 0.96	6.66 ± 3.25	ns
Ratio of monocytes/lymphocytes (M%/L%), median (IQR)	0.18 (0.15, 0.21)	0.48 (0.22, 0.74)	****

*** P-value is <0.001; **** P-value is <0.0001.

ns, not significant.

### The mRNA expression of Notch1, DLL1 and Hes1 were upregulated in PBMCs of TB patients compared to uninfected individuals

**Figure 1:**
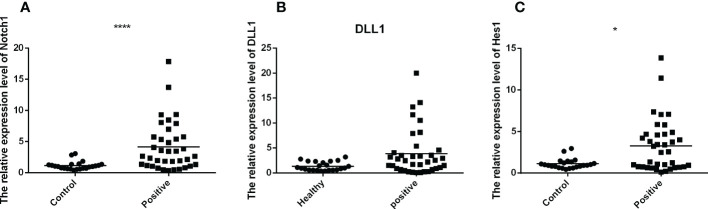
The mRNA expression of Notch1, DLL1 and Hes1 of active TB patients (n=37) and healthy controls (*n*=23) were detected by RT-QPCR. The data were standardized to β-actin and presented as the mean ± SD of triple independent experiments. Mann-Whitney U test (Wilcoxon rank sum test) was used for statistical analysis. **(A–C)** respectively showed that the mRNA expression of Notch1, DLL1, and Hes1. *p < 0.05, ****p < 0.0001.

To investigate the mRNA expression of Notch1, DLL1 and Hes1 in PBMCs of TB-infected and uninfected individuals, Q-PCR was performed for comparison. It was observed that the mRNA expression of Notch1, DLL1 and Hes1 were upregulated in the PBMCs of TB patients compared to uninfected individuals. (**Figures 1A–C**). These findings demonstrated that the expression of Notch pathway molecules was up-regulated during TB infection.

### The levels of Notch signaling molecules were higher in moderate/severe TB patients than that in mild and uninfected individuals

**Figure 2:**
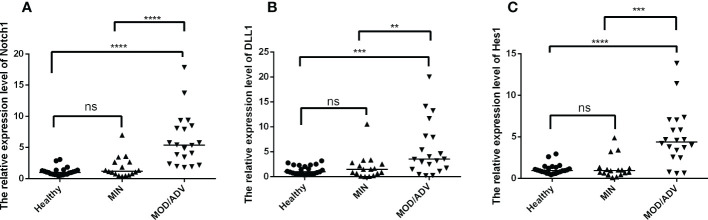
Comparison of the mRNA expression of Notch1, DLL1 and Hes1 in healthy individuals (*n*=23) and active TB patients with mild (n=17) or moderate/severe (n=20) disease were compared by using Kruskal-Wallis one-way analysis of variance by ranks. The standardization of data were used the housekeeping gene β-actin and shown as the mean ± SD of three times experiments. **(A–C)** presented the mRNA expression of Notch1, DLL1, and Hes1 in healthy controls and patients with different degrees of disease, respectively. **p < 0.01, ***p < 0.001, ****p < 0.0001, *ns*, not significant.

To further explore the levels of Notch signaling molecules at different stages of TB infection, participants were divided into healthy (n=23), mild (n=17), and moderate/severe (n=20) groups based on the lung injury and symptoms meeting the diagnosis criteria of Tuberculosis WS288-2017. As shown in **Figures 2A–C**, the mRNA expression of Notch1, DLL1 and Hes1 were significantly higher in patients with moderate/severe TB compared to those with mild TB or no infection. However, there was no difference observed between the mild and healthy participants. These results suggested that the expression of Notch signaling molecules may be associated with the severity of TB infection.

### The mRNA expression of T-bet and IFN-γ were down-regulated in PBMCs of TB patients compared to uninfected individuals

**Figure 3:**
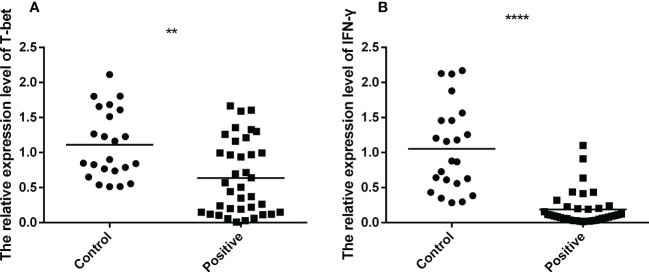
The mRNA expression of T-bet and IFN-γ of active TB patients (*n*=37) and healthy controls (*n*=23) were tested by RT-QPCR. The data were normalized quotient to β-actin and presented as the mean ± SD of three independent tests, performed by Mann-Whitney U test (Wilcoxon rank sum test). **(A, B)**, respectively, shown as the mRNA expression of T-bet and IFN-γ. **p < 0.01, ****p < 0.0001.

To explore the mRNA expression of T-bet and IFN-γ in PBMCs of TB infected and uninfected individuals, Q-PCR was performed for comparison. It was observed that the mRNA expression of T-bet and IFN-γ were down-regulated in the PBMCs of TB patients compared to uninfected individuals (**Figures 3A, B**). These results demonstrated that the expression of Th1-type factors were down-regulated during TB infection.

### The levels of Th1-type factors were lower in mild and moderate/severe TB patients than that in uninfected individuals

**Figure 4:**
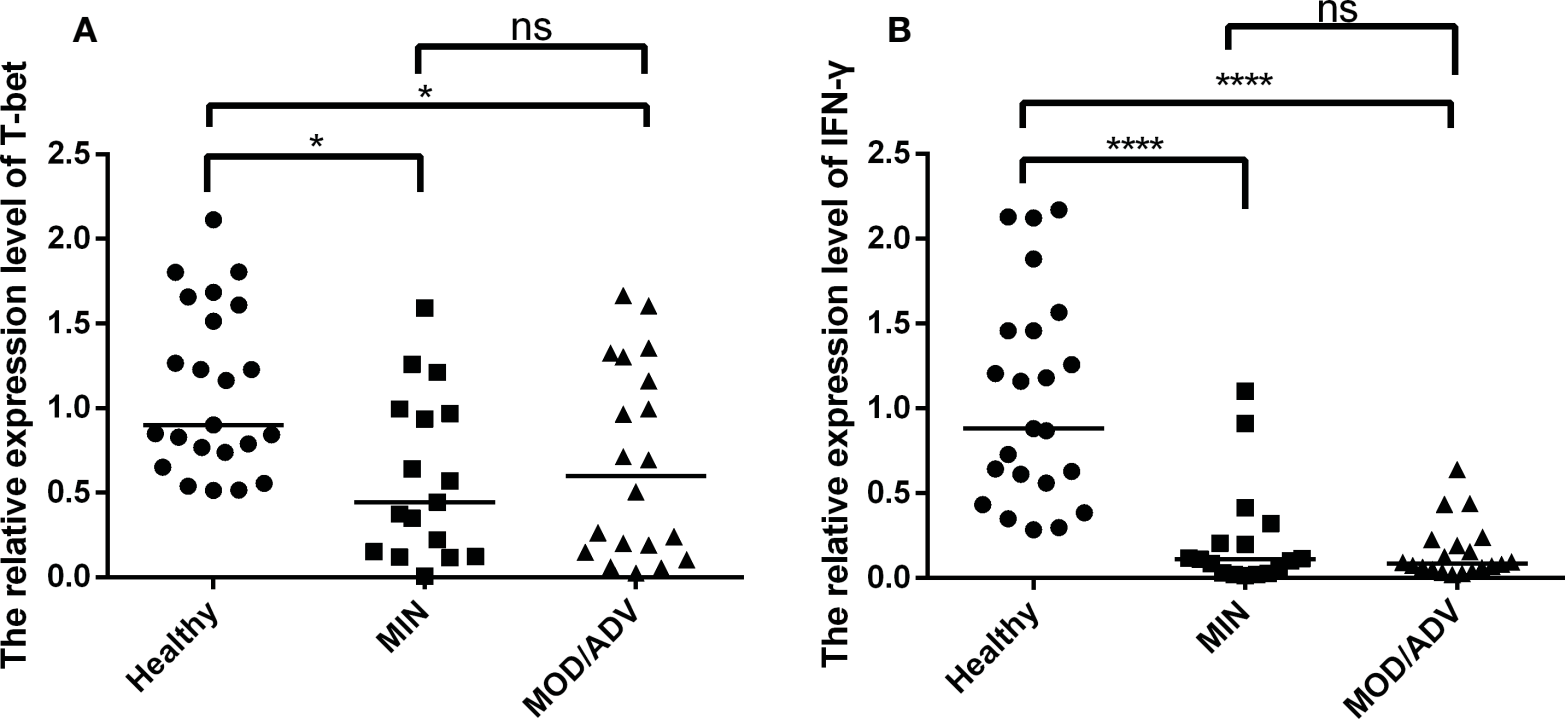
Comparison of the mRNA expression of T-bet and IFN-γ in healthy (*n*=23), mild (*n*=17) and moderate/severe (*n*=20) patients, performed by Kruskal-Wallis’s test. The normalization of data were used the internal control gene β-actin and presented as the mean ± SD of three technical replicates. **(A, B)**, respectively, show the mRNA expression of T-bet and IFN-γ in different individuals groups. *p < 0.05, ****p < 0.0001, *ns*, not significant.

To further explore the levels of T-bet and IFN-γ at different stages of TB infection, participants were divided into healthy, mild and moderate/severe groups (the number of participators respectively were 23, 17, 20) based on the lung injury and symptoms that met the diagnosis criteria of Tuberculosis WS288-2017. As shown in **Figures 4A, B**, the mRNA expression of T-bet and IFN-γ were lower in mild and moderate/severe TB patients than that in uninfected individuals and the difference were statistically significant. No difference was observed between the mild and moderate/severe groups. These results suggested that a decrease in the frequency of Th1 cells, and the immune response mediated by Th1-type factors is tends to be impaired in the progression of TB patients.

### The combiROC of IFN-γ and L% contribute to discriminate the active TB patients from healthy individuals, while the combiROC of Notch1 and Hes1 was favored for distinguishing mild TB patients from moderate/severe individuals

**Figure 5:**
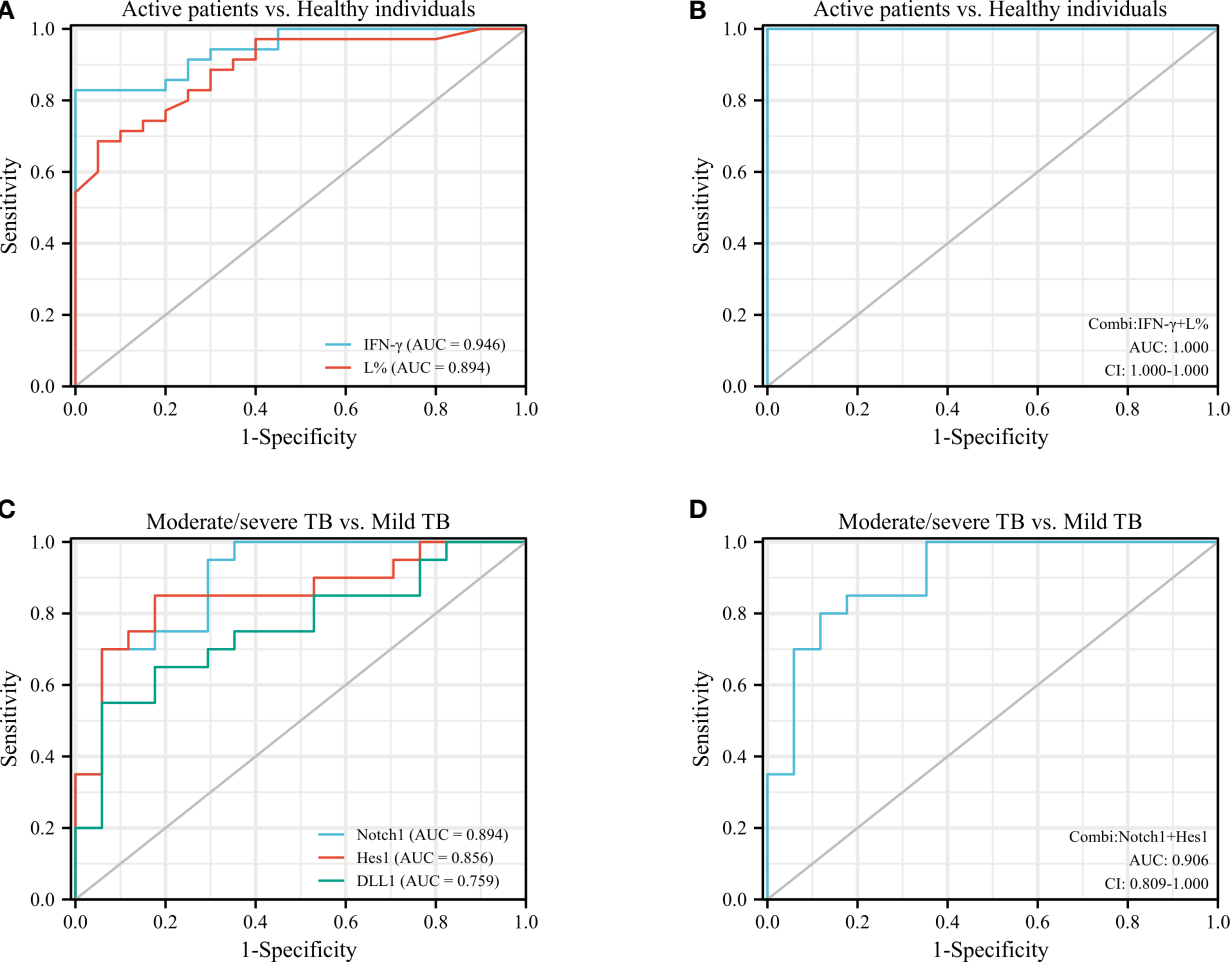
ROC analysis was performed to assess the diagnostic value of IFN-γ, L%, Notch1, DLL1, and Hes1 in patients with different stages of active TB. **(A)**, ROC analysis of IFN-γ and L% in active TB cases vs. healthy individuals; **(B)**, combiROC of IFN-γ and L% in active TB patients vs. healthy controls; **(C)**, ROC analysis of Notch1, DLL1 and Hes1 in moderate/severe TB patients vs. mild TB patients; **(D)**, combiROC analysis of Notch1 and Hes1 in moderate/severe TB cases vs. mild TB cases.

The results presented above proved that the Notch signaling molecules and Th1-type factors had changed differently at different stages of TB patients, as well as the WBC parameters. To further verify the diagnostic efficiency of Notch1, DLL1, Hes1, T-bet, IFN-γ and the percentage of lymphocytes (L%) in discriminating the severity of the disease provoked by TB from healthy individuals, the ROC analysis method was used (**Figure 5**).

The results showed that IFN-γ and L% were more effective than other indicators in aiding the diagnosis of active or inactive TB, with an AUC of 0.946 and a cut-off point of 0.26 for IFN-γ, and an AUC of 0.894 and a cut-off point of 20.95 for L% (**Figure 5A**). And then the accuracy of their predictions was achieved the best diagnostic efficacy in the combined model of both (**Figure 5B**), which was with the AUC value of 1.00 and cut-off point of 0.136. On the other hand, the molecules of the Notch pathway (Notch1, DLL1 and Hes1) were superior to the factors of Th1 cells (T-bet and IFN-γ) and the WBC parameters in terms of diagnostic efficacy for discriminating mild from moderate/severe TB, with AUC values of 0.897, 0.853 and 0.753, for Notch1, DLL1, and Hes1, respectively (**Figure 5C**). It is worth noting that the diagnostic efficacy of Notch1 and Hes1 was close, with an AUC value of 0.906 and cut-off point of 0.066 for the combined model (**Figure 5D**). And a sensitivity of 0.800, specificity of 0.882, and CI in the range of 0.809-1.000, this model has high accuracy in discriminating mild from moderate/severe TB patients.

Consequently, the diagnostic efficiency of the combined ROC analysis of IFN-γ and L% had the highest accuracy in discriminating the active TB patients from healthy individuals. Additionally, the combiROC of Notch1 and Hes1 was favored for distinguishing mild TB patients from moderate/severe individuals. These findings highlight the potential diagnostic utility of Notch pathway molecules as biomarkers for management active TB, particularly in identifying disease severity.

## Discussion

Despite advancements in TB prevention and treatment, the disease remains a significant global health threat with high morbidity and mortality rates. Hyperreactive TB can cause chronic inflammation, leading to irreversible damage in older adults with subtle symptoms. Post-TB sequelae can significantly add to the overall burden of pulmonary TB by reducing lung function and quality of life (19). Early diagnosis and monitoring of TB progression can decrease the risk of long-term sequelae. Identifying biomarkers to monitor TB progression, including post-TB, is clinically significant.

This study analyzed the expression of Notch pathway molecules (Notch1, DLL1 and Hes1) and Th1-type response factors (T-bet and IFN-γ) in TB patients, along with routine WBC parameters. The results of WBC parameters showed a significant reduction of lymphocytes and a corresponding increase in the proportion of neutrophils during active TB progression. This phenomenon is typically seen in chronic bacterial infections or mild viral infections. It has been reported that an elevated neutrophils ratio is significantly associated with a decline in lung function, while the opposite is true for the lymphocytes frequency (20). Additionally, the results of lymphocytes were consistent with findings from previous studies indicating their crucial role in mycobacterial clearance (17, 18). Other report also considered that the frequency of lymphocytes in peripheral blood is one of the valuable parameters for predicting the risk of active TB (21). Therefore, monitoring the frequency of lymphocytes in peripheral blood is particularly important during the progression of active TB.

The results of this study confirmed that the mRNA expression of Notch1, DLL1 and Hes1 were higher in TB group compared to the control group, particularly in patients with moderate/severe disease. These results were consistent with previous researches (12, 14), indicating that the Notch signaling pathway is aberrantly activated in TB patients with moderate/severe. In contrast, the expression of Th1-type factors (T-bet and IFN-γ) were down-regulation compared to the healthy controls, with no significant difference between mild and moderate/severe patients. These results concurred with previous studies (22, 23), but rather, they contradicted earlier studies (24, 25). It may be attributed to the fact that earlier studies mostly focus on the transitional response between latency and activity. In the early stages of TB infection, Th1 cells activation and IFN-γ secretion play an immunoprotected role (2, 3). But as the disease proceeds, there is a severe depletion of T cells and suppression of innate and adaptive immune function, particularly the reduction of IFN-γ-secreting Th1 cells (22). This suppression is accompanied by an increase in other pro-inflammatory cells and cytokines (23, 26, 27), factors accelerate the progression of TB patients to severe disease. The reduction of Th1 response is associated with poor prognosis in patients with TB infection (28). In this study, the decreased frequency of Th1 cells may predict that Th1-type-mediated protective immune responses tend to be impaired as TB disease progress. This requires further *in vitro* validation or disease follow-up studies. Meanwhile, this highlights the need for new treatments for TB that boost Th1 immune responses. On the other hand, we further analyzed the correlation between Notch signaling molecules expression and the WBC parameters, as well as the Th1-type factors, but no correlation was found (data not shown). Other complex mechanisms may regulate the Notch pathway and WBC in blood separately. However, the Notch pathway may still be involved in regulating other pro-inflammatory cells or pathways that impact the TB infection process. Although the literature on this topic is scarce and inconclusive, our findings suggest that further exploration in this area is necessary.

Remarkably, there have been new discoveries of Notch signaling molecules in disease treatment. Several studies have shown that therapeutic targeting of molecules in the Notch pathway can be effective in the prevention and diseases treatment (29–31). Thus, it is clear that the Notch signaling is involved in cell differentiation and offers new ideas for the treatment of some inflammatory diseases. However, less research has been reported on its usefulness as a biomarker for monitoring disease progression. In this paper, the potential monitoring efficacy of Notch signaling molecules was evaluated using ROC curves, along with Th1-type response-related factors and routine WBC parameters at different stages of TB disease progression.

The results showed that both the L% of WBC parameters and the level of IFN-γ were superior to other genes in aiding the diagnosing active TB. The combination of both was the most effective model. Currently, IFN-γ is widely used as an aid in monitoring the presence or absence of TB infection. However, it is not yet ideal for monitoring the progression of TB disease. Previous studies (18, 21) have also found that lymphocyte parameters, such as L%, can be used to predict the risk of active TB disease. ​At present, the application of the lymphocyte parameter L% in the monitoring active TB is not mentioned in the domestic diagnostic criteria (15). This study showed that the combined model of IFN-γ and L% was helpful in identifying individuals with active TB. The blood cell count is widely used in clinical practice, easy to operate and inexpensive. Therefore, Combining IFN-γ and L% may aid in the diagnosis of active TB. This may benefit more patients, especially in suspicious cases with negative sputum test for bacteria or nucleic acid of the pathogenic bacteria.

On the other hand, the study found that Notch1, DLL1, and Hes1 molecules exhibit superior diagnostic efficacy in discriminating mild from moderate/severe TB patients compared to Th1-type factors and routine WBC parameters. The combined model of Notch1 and Hes1 had high accuracy in diagnostic efficacy. These findings suggest that Notch1 and Hes1 molecules are potential biomarkers for mild and moderate/severe TB, providing a basis for adjunctive diagnosis and monitoring of disease efficacy. Previous research has identified Notch3 as a diagnostic marker for cerebral autosomal dominant arteriopathy with subcortical infarcts and leukoencephalopathy (CADASIL) (32, 33), while DLL1 has shown utility in diagnosing tuberculous meningitis (12). Although no other Notch signaling molecules have been identified as biomarkers for other diseases, the regulation of immune cells function and cytokines secretion by Notch signaling molecules can affect the outcome of TB disease. Consequently, using Notch signaling molecules as biomarkers of TB disease progression can provide a more accurate reflection of the direction of disease progression. This paper proposes for the first time that Notch1 and Hes1 can be used as potential biomarkers to assist in the monitoring of tuberculosis-induced moderate/severe lung disease, providing novel ideas and directions to support clinical monitoring and therapeutic effect observation.

In addition, there are still several limitations of this study. Firstly, more studies are needed to support the potential clinical monitoring value, including more data on the comparison of monitoring efficacy from randomized trials containing patients of different ages and with complex TB-associated disease. Also, pre- and post- treatment testing of different treatment regimens. Secondly, the expression of Notch1 and Hes1 should be tested in post- treatment of patients with TB infection, which would provide comprehensive data for a longitudinal study of Notch1 signaling. Finally, more comprehensive studies are needed to fully understand the function of Th1 cells and the mechanisms between Notch signaling pathway and other cellular and factor alterations of immune responses in active TB patients.

## Data availability statement

The raw data supporting the conclusions of this article will be made available by the authors, without undue reservation.

## Ethics statement

This study involving human participants was reviewed and approved by The Ethics Committee of Xinhui District People’s Hospital affiliated with Southern Medical University, Jiangmen. The patients/participants provided their written informed consent to participate in this study.

## Author contributions

This project was designed and directed by GL and JX. JX, YC, SC, WZ and HL conducted the data of participators collection and analysis, as well as the experimental data. The manuscript was written by GL and JX. All authors contributed to the article and approved the submitted version.
